# Early Recognition of Atypical Thrombotic Thrombocytopenic Purpura (TTP) in Severe Asymptomatic Thrombocytopenia Using the PLASMIC Score and Peripheral Blood Smear

**DOI:** 10.7759/cureus.84676

**Published:** 2025-05-23

**Authors:** Khiet T Nguyen, Kaung H Win, Giao M Tran, Dat Nguyen, Hau Nguyen

**Affiliations:** 1 Medicine, Interfaith Medical Center, New York, USA; 2 Internal Medicine, Interfaith Medical Center, New York, USA; 3 Geriatrics, Nhan Dan Gia Dinh Hospital, Ho Chi Minh City, VNM; 4 Interventional Cardiology, Nhan Dan Gia Dinh Hospital, Ho Chi Minh City, VNM; 5 Cardiac Surgery, Cho Ray Hospital, Ho Chi Minh City, VNM

**Keywords:** asymptomatic severe thrombocytopenia, immune ttp, peripheral blood smear, plasmic score, therapeutic plasma exchange (tpe)

## Abstract

Thrombotic thrombocytopenic purpura (TTP) is the clinical syndrome of cluster symptoms of thrombotic microangiopathy, which rarely manifests with all classic symptoms of fever, neurological changes, acute kidney failure, hemolytic anemia, and thrombocytopenia. This case describes a patient who was admitted for acute abdominal pain, initially suspected to be pancreatitis, and found to have severe, asymptomatic thrombocytopenia. The diagnostic challenge was compounded by overlapping factors, including chronic alcohol use and vitamin B12 deficiency. A comprehensive workup, including PLASMIC score (platelet count, hemolysis, absence of active cancer, absence of solid organ or stem cell transplant, mean corpuscular volume, international normalized ratio, and creatinine) assessment and peripheral blood smear, ultimately led to the diagnosis of acquired immune TTP (iTTP). Early recognition enabled timely treatment and likely prevented severe complications.

## Introduction

Thrombotic thrombocytopenic purpura (TTP) was previously characterized as a rare, life-threatening condition with thrombotic microangiopathy events, biologically marked by bleeding, hemolytic anemia, and severe thrombocytopenia, and clinically presenting with ischemic symptoms of the brain, kidneys, and other visceral organs. The pathology of this syndrome is the deficiency of ADAMTS13 enzyme activity, which could be inherited from congenital conditions due to hundreds of gene mutations or acquired by forming ADAMTS13 antibodies known as immune TTP or iTTP, which reduces the ability to prevent the blood accumulation of platelet-hyper adhesive ultra-large von Willebrand factor (VWF) multimers, leading to the development of clots composed primarily of platelets in the microvasculature [1].

The full clinical pentad of TTP with fever, neurological symptoms, thrombocytopenia, hemolytic anemia, and renal dysfunction, which is seen in less than 10% of cases, makes early diagnosis difficult. Prompt recognition is critical, as untreated cases carry a mortality risk approaching 90%, while timely treatment significantly improves survival [2].

## Case presentation

A 40-year-old female with a history of hypertension (poorly controlled due to nonadherence to labetalol), chronic alcohol use, came to the emergency department with epigastric pain of three days’ duration. The pain was described as a burning sensation radiating to the midsternal area, intermittent in nature, and began following a two-day binge of alcohol over the weekend. It was associated with nausea, non-bloody emesis containing food particles, and decreased oral intake.

She denied lower abdominal pain, chest pressure, shortness of breath, bowel or urinary symptoms, fever, cough, trauma, or a personal history of hematologic disorders. On physical examination, no hepatosplenomegaly, petechiae, or signs of bleeding were noted. The only positive finding was mild tenderness in the epigastric region on deep palpation.

She was admitted to the medical floor for management of suspected acute pancreatitis, likely alcohol-induced, versus alcohol-induced gastritis. Labs on arrival are shown in Table 1. Abdominal ultrasound showed no gallstones or hepatomegaly and was otherwise unremarkable.

**Table 1:** Laboratory Results on the Day of Arrival WBC: White blood cell count; MCV: Mean corpuscular volume; MCH: Mean corpuscular hemoglobin; PLT: Platelet count; EDTA: Ethylenediaminetetraacetic acid; INR: International normalized ratio; aPTT: Activated partial thromboplastin time; BUN: Blood urea nitrogen; eGFR: Estimated glomerular filtration rate; AST: Aspartate aminotransferase; ALT: Alanine aminotransferase

Laboratory Test (Day of Arrival)	Result	Reference Range
WBC	10.8 × 10³/μL	4.5-11.0 × 10³/μL
Hemoglobin	13.3 g/dL	11.0-15.0 g/dL
MCV	95.2 fL	80-100 fL
MCH	31.8 pg	26.0-33.0 pg
PLT	7 × 10³/μL	130-400 × 10³/μL
Platelet count with EDTA tube	5.5 × 10³/μL	130-400 × 10³/μL
Prothrombin time	11.5 sec	9.8-13.4 sec
INR	0.97	0.85-1.15
aPTT	31.7 sec	24.9-35.9 sec
BUN	16 mg/dL	7-25 mg/dL
Creatinine	0.8 mg/dL	0.6-1.2 mg/dL
eGFR	95.5 mL/min/1.73m²	≥90.0 mL/min/1.73m²
AST	34 U/L	13-39 U/L
ALT	26 U/L	7-52 U/L
Lipase	286 U/L	11-86 U/L
Albumin	4.1 g/dL	3.4-5.4 g/dL
Total protein	6.7 g/dL	6.0-8.3 g/dL

Initial laboratory workup revealed severe thrombocytopenia with a platelet count of 7 × 10^3^/μL (reference: 130-400 × 10^3^/μL). A repeat count using an ethylenediaminetetraacetic acid (EDTA) tube confirmed 5.5 × 10^3^/μL. Her last recorded platelet count one year prior was 200 × 10^3^/μL. Despite the absence of active bleeding and a normal hemoglobin level of 13.3 g/dL, she received one unit of platelet transfusion in the emergency department.

Further workup showed negative serologies for human immunodeficiency virus (HIV), hepatitis B virus (HBV), and hepatitis C virus (HCV), and a borderline low vitamin B12 level at 282 pg/mL (normal range: 180-914 pg/mL). Initial management focused on severe thrombocytopenia, possibly due to vitamin B12 deficiency or bone marrow suppression from chronic alcohol use. She was started on vitamin B12 injections (1000 mcg daily), dexamethasone 20 mg IV every eight hours for three days, and intravenous immunoglobulin (IVIG) 400 mg/kg/day.

**Figure 1:**
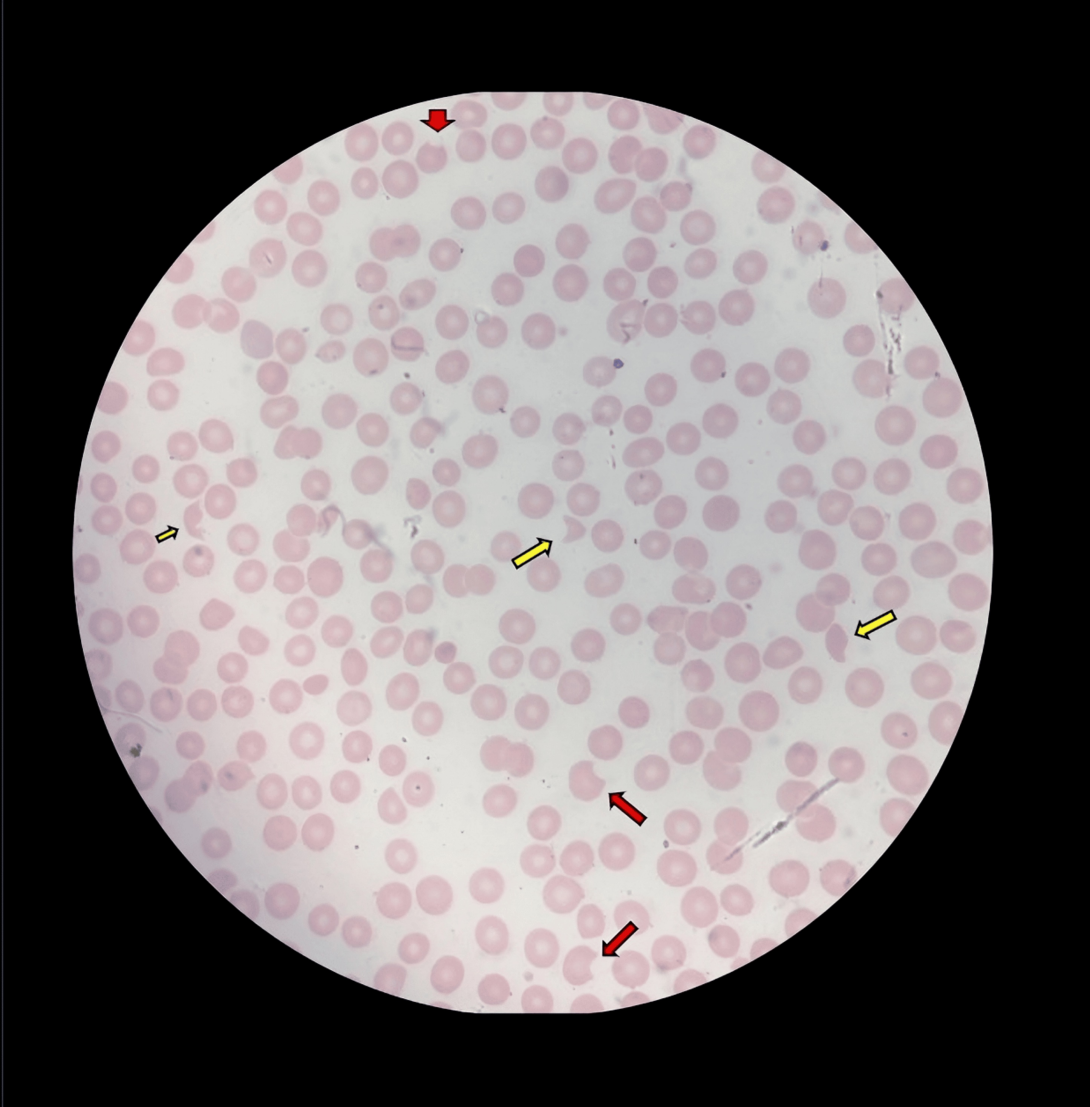
Peripheral Blood Smear (PBS) PBS showed bite cells (red arrows) and fragmented red blood cells, including helmet cells (yellow arrows), viewed under 100x oil immersion.

A bone marrow biopsy was planned due to persistent thrombocytopenia after three days of treatment. Peripheral blood smear revealed schistocytes, fragmented red blood cells, and helmet cells, with less than 1% bite cells observed in Figure 1.

Given the clinical concern for thrombotic microangiopathy, a hemolytic anemia workup was performed despite the patient’s hemoglobin remaining within the normal range (12-13.3 g/dL). Laboratory evaluation revealed an elevated lactate dehydrogenase (LDH) level of 705 U/L (reference: 140-271 U/L), markedly reduced haptoglobin <10 mg/dL (reference: 33-278 mg/dL), total bilirubin of 2.1 mg/dL with direct bilirubin of 0.28 mg/dL, and a negative direct Coombs test findings are supportive of non-immune hemolytic anemia.

Due to high suspicion for TTP, ADAMTS13 activity and antibody tests were sent early in the hospital course, but were pending due to delayed processing. After five days of treatment, platelet counts remained critically low. Results later returned with ADAMTS13 enzyme activity of 2.3% (reference: >66.8%) and ADAMTS13 antibody level of 13 U/mL (reference: <12 U/mL), confirming a diagnosis of iTTP.

**Figure 2:**
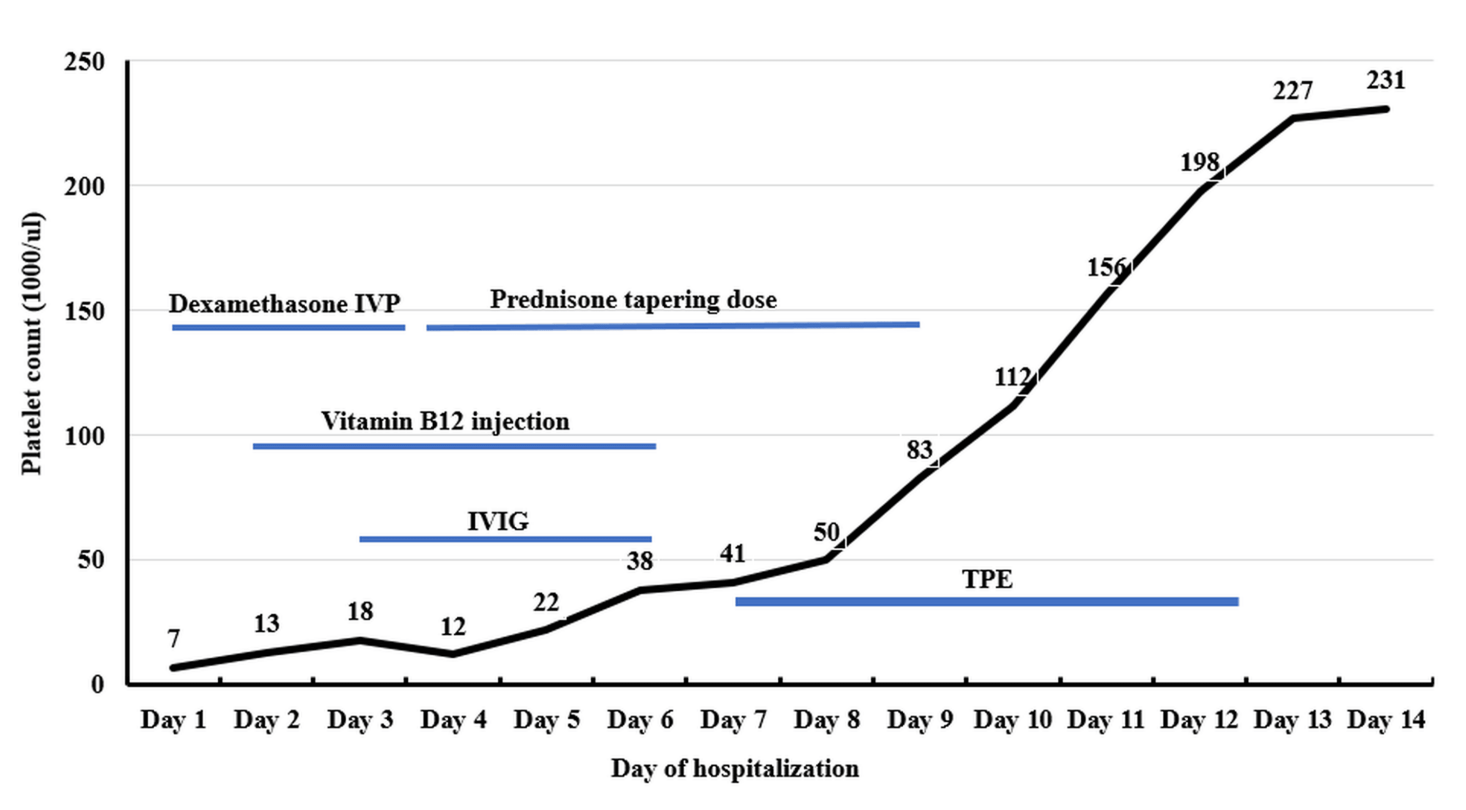
Timeline of Platelet Count Recovery and Therapeutic Interventions IVP: Intravenous push; IVIG: Intravenous immunoglobulin; TPE: Therapeutic plasma exchange

The patient was transferred to the ICU and started on plasmapheresis via a femoral central venous catheter. Platelet counts normalized after four sessions of therapeutic plasma exchange (TPE), as illustrated in Figure 2. Bone marrow biopsy subsequently reported hypercellular marrow with maturing trilineage hematopoiesis, with no evidence of acute leukemia, metastatic neoplasm, plasma cell dyscrasias, or lymphoma, findings supportive of TTP; however, the histopathological images were not able to be accessed from a third-party company.

## Discussion

Since the first fatal case of TTP was described in 1924, the condition has been characterized by systemic microvascular thrombosis, causing transient neurological changes, fever, hemolytic anemia, and severe thrombocytopenia. Until the late 1990s, TTP was associated with a mortality rate approaching 90% due to delays in recognition and limited therapeutic options.

A breakthrough came in 1985, when histopathologic studies of deceased TTP patients revealed widespread platelet-rich thrombi containing unusually large VWF multimers, rather than fibrin. This discovery led to the adoption of TPE as a therapeutic strategy, which has since become the cornerstone of treatment, improving survival rates to over 85% [1].

More recently, a severe deficiency in ADAMTS13 enzyme activity (<10%) has been established as the diagnostic hallmark of TTP. However, since ADAMTS13 testing often requires two to three days for results, clinical scoring systems like the PLASMIC score (platelet count, hemolysis, absence of active cancer, absence of solid organ or stem cell transplant, mean corpuscular volume, international normalized ratio, and creatinine) are now routinely used to assess pretest probability and guide early empiric treatment.

In this case, the patient presented with severe asymptomatic thrombocytopenia (7 × 10^3^/μL), confirmed on repeat CBC using EDTA anticoagulant (5.5 × 10^3^/μL). Common secondary causes of thrombocytopenia were ruled out (negative HIV, HBV, and HCV), though the patient was found to have borderline vitamin B12 deficiency (282 pg/mL) and a significant history of chronic alcohol use. In the absence of splenomegaly on physical exam and imaging, alcohol-induced bone marrow suppression was considered a potential contributor [3,4].

Initial treatment included vitamin B12 injections, corticosteroids, IVIG, and a unit of platelet transfusion given in the emergency department. Although platelet transfusion is generally avoided in suspected TTP due to the risk of exacerbating thrombotic events [5], data remain inconclusive, and harm has not been definitively proven in all cases [6,7]. Given the uncertain diagnosis at that time, the benefits of supportive therapy (IV corticosteroid with IVIG along with administration of vitamin B12) were felt to outweigh the risks.

After five days of therapy, the patient’s platelet count remained below 50 × 10^3^/μL. A bone marrow biopsy was performed to investigate other potential hematologic causes, and a peripheral blood smear revealed helmet cells and schistocytes (<1%), though not in significant numbers. Despite a normal hemoglobin level (13.2 g/dL), further evaluation revealed laboratory features of non-immune hemolysis: LDH 705 U/L, haptoglobin <10 mg/dL, total bilirubin 2.1 mg/dL, direct bilirubin 0.28 mg/dL, and a negative direct Coombs test.

A PLASMIC score of 6 was calculated based on the following criteria: platelet count less than 30,000/μL; evidence of hemolysis; absence of active cancer; no history of solid organ or stem cell transplant; international normalized ratio (INR) less than 1.5; and creatinine level below 2.0 mg/dL.

This suggested a high probability of TTP [8]. It is worth noting that although the PLASMIC score includes hemolysis-related criteria, peripheral blood smear findings are not incorporated into the scoring system. ADAMTS13 activity and antibody testing were sent at the time of admission, but results were delayed. On hospital day 6, the patient was transferred to the ICU, and TPE was initiated via femoral vein catheterization once plasma products became available.

It is noteworthy that the patient’s platelet count remained stagnant during initial corticosteroid and IVIG therapy, only rising dramatically after three days of TPE, ultimately reaching normal levels. Some studies suggest that high-dose IVIG may be effective as salvage therapy in TTP refractory to steroids and TPE [9], and others have proposed IVIG as a potential first-line therapy in resource-limited settings due to its relative cost-effectiveness [10].

In our case, however, TPE proved to be the most effective intervention, supporting its continued role as the gold standard. Nonetheless, recent reports exploring TPE-free approaches using immunosuppressive regimens raise important questions about the evolving management of iTTP [2], particularly in facilities where TPE is not readily available.

## Conclusions

TTP is a rare but potentially fatal condition with a high mortality rate if left untreated. Early recognition remains challenging, as the classic pentad - neurological changes, fever, hemolytic anemia, renal injury, and thrombocytopenia - is often incomplete at initial presentation. In some cases, severe isolated thrombocytopenia may be the only early finding. The use of the PLASMIC score and peripheral blood smear can provide critical guidance for early diagnosis and initiation of treatment while awaiting confirmatory ADAMTS13 testing. Although TPE remains the cornerstone of treatment, emerging evidence suggests that immunosuppressive therapies may offer effective alternatives in selected patients, particularly where TPE access is limited.
